# Acetylsalicylic acid aggravates anaphylaxis in a PGE_2_-dependent manner

**DOI:** 10.1172/JCI175397

**Published:** 2025-03-03

**Authors:** Philipp Globig, Payam Morakabati, Veronika Höfer, Diana M. Willmes, Magda Babina, Margitta Worm

**Affiliations:** 1 Division of Allergy and Immunology, Department of Dermatology, Venereology and Allergology, Charité – Universitätsmedizin Berlin, Berlin, Germany.; 2 Institute of Allergology, Charité – Universitätsmedizin Berlin, Corporate Member of Freie Universität Berlin and Humboldt-Universität zu Berlin, Berlin, Germany.; 3 Fraunhofer Institute for Translational Medicine and Pharmacology ITMP, Immunology and Allergology IA, Berlin, Germany.

**Keywords:** Inflammation, Allergy, Mast cells

## Abstract

Acetylsalicylic acid (ASA) can exert proanaphylactic effects, but the extent of this phenomenon and its underlying mechanisms are undefined. Yet, low homeostatic prostaglandin E_2_ (PGE_2_) levels have been associated with anaphylaxis. In this study, we investigated whether the proanaphylactic effect of ASA is PGE_2_ dependent. We assessed the effect of ASA in experimental anaphylaxis models, analyzed a large dataset of patients with anaphylaxis, and performed titrated allergen challenges in ASA-treated allergic individuals. Registry data indicated an increased risk for severe anaphylaxis in patients with ASA comedication. ASA pretreatment aggravated allergen-dependent anaphylaxis in mice, whereas histamine-induced anaphylaxis remained unaffected. Exacerbation was due to reduced PGE_2_, as its stabilization or the use of prostanoid E receptor (EP) agonists reversed the proanaphylactic effects of ASA. EP2-, EP3-, and EP4 receptor–deficient mice revealed that each receptor individually contributed to ASA susceptibility. In patients with allergy, prior ASA intake increased skin responsiveness to allergen but not to histamine. Conversely, the responses of basophils to ex vivo FcεRI aggregation remained unaltered, indicating that ASA operated by enhancing the stimulability of mast cells in a PGE_2_-dependent manner. Collectively, our data reveal a central role of the PGE_2_ network in ASA-aggravated anaphylaxis. EP receptors could be potential targets to prevent or alter the outcome of anaphylaxis.

## Abstract 2

ASA pretreatment aggravated allergen-dependent anaphylaxis in mice. Exacerbation was due to reduced PGE2, since receptor agonists reversed ASA’s pro-anaphylactic effects.

## Introduction

Anaphylaxis is a severe and potentially life-threatening hypersensitivity reaction. In type 1 allergy, it results from the interaction between IgE-binding allergens (1–4), leading to mast cell (MC) and basophil activation and the release of numerous mediators eliciting the clinical reaction. Anaphylaxis can also be triggered in an IgE-independent manner, e.g., through the activation of the Mas-related GPCR X2 (MRGPRX2) with certain drugs, the complement system, or even IgG (5). Many cofactors have been reported to modulate the onset, but also the severity, of anaphylaxis (6, 7). Besides intrinsic factors like sex or higher age, extrinsic factors such as physical exercise or drugs like NSAIDs have been reported as cofactors in up to 40% of anaphylaxis occurrences (8).

Acetylsalicylic acid (ASA) is a frequently applied NSAID representative. As an inhibitor of cyclo-oxygenases 1 and 2 (COX1, COX2), also known as prostaglandin (PG) synthetases 1 and 2, ASA suppresses the production of prostaglandins (9, 10). Prostaglandins play a role in the progression of fever, pain, and inflammation (11), and their inhibition explains the analgesic, antipyretic, and antiinflammatory properties of ASA. Previous studies suggested that the proanaphylactic effect of NSAIDs in food allergy reactions may arise from COX inhibition, with a subsequent reduction in PG levels (12–14).

We recently demonstrated a potential protective role of prostaglandin E_2_ (PGE_2_) in anaphylaxis, with patients at risk of anaphylaxis having lower baseline PGE_2_ levels compared with controls (15). We therefore hypothesized that ASA may affect anaphylaxis by modulating the bioavailable PGE_2_.

PGE_2_ is the most abundant PG subtype. It exhibits a complex mode of action with a wide range of (sometimes opposing) biological effects on both physiology and pathology (16–19). The diversity and complexity of its biological effects are created at distinct levels and have no counterpart in any other prostanoid signaling systems. PGE_2_ is controlled at the level of PGE_2_ synthesis with 3 specific synthases (9) and acts through four PGE_2_ receptors (EP1–EP4), which are all members of the GPCR family (17).

In this study, we analyzed whether and by what mechanism ASA influences anaphylaxis. For this purpose, we applied several in vivo and in vitro approaches to delineate the role of PGE_2_ and its receptors in this condition. Our data reveal an aggravating effect of ASA on anaphylaxis in humans and mice, thus providing evidence of the central role of PGE_2_ in anaphylaxis, and suggest EP receptor activation as a potential target to alter the outcome of anaphylaxis.

## Results

### Acetylsalicylic acid is associated with increased anaphylaxis severity in humans.

**Figure 1:**
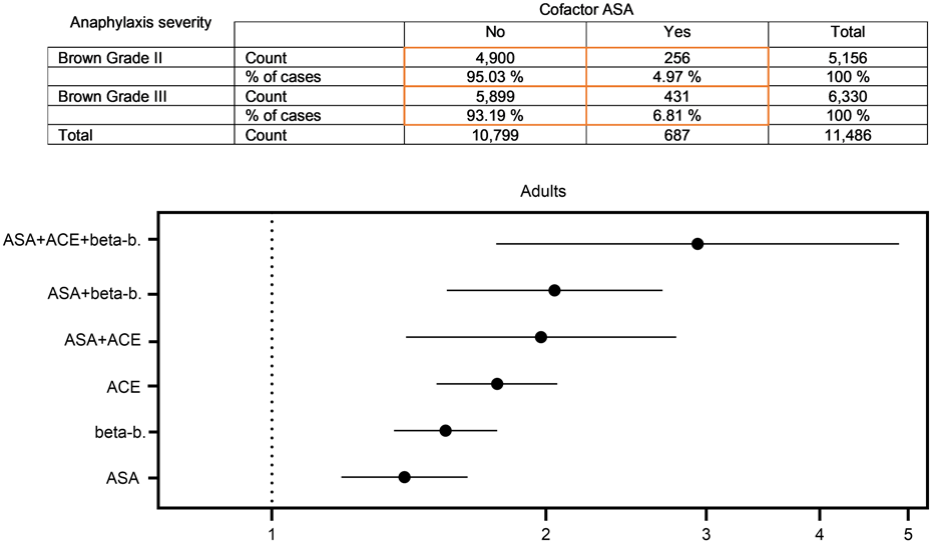
Comparison of the severity of anaphylaxis in adult patients, with or without the cofactor ASA with calculated ORs. OR, 95% CI. beta-b, beta blocker.

To assess whether ASA may affect the severity of anaphylaxis in humans, we analyzed data from the European Anaphylaxis Registry. This is a large data source in which cases of anaphylaxis in patients are reported by specialized allergy centers in Europe and Brazil (grades 2–4 according to Ring and Messmer). Up to now, a total of 18,948 patients have been registered. Considering adults with a reaction of grades 2 and 3 according to Brown’s severity grading, a total of 687 cases among 11,486 cases were identified as having reported concomitant intake of ASA in the context of an anaphylactic reaction. The proportion of ASA intake was higher in the group of patients who experienced a more severe reaction (*n* = 256, Brown grade 2 [4.97%] vs. *n* = 431, Brown grade 3 [6.81%]). The crude OR revealed an increased risk for a more severe reaction (grade 3) in adults compared with moderate reactions (grade 2) with ASA as a cofactor (1.398 [1.192 – 1.640]). We previously described that the intake of beta blockers and/or angiotensin-converting enzyme (ACE) inhibitors increases the risk for severe anaphylaxis (7). In real life, many older patients with cardiovascular conditions receive triple therapy consisting of beta blockers, ACE inhibitors, and ASA. We therefore determined the OR for severe reactions in the different therapy regimens. Indeed, we found an increased risk for severe reactions when ASA was taken together with a beta blocker (2.044 [1.556–2.685]) or an ACE inhibitor (1.975 [1.403–2.781]). The highest risk for a more severe reaction was determined when all 3 medications were taken by patients simultaneously (2.935 [1.763–4.886]) (Figure 1).

To analyze whether ASA intake has a robust effect on the severity of anaphylaxis, we adjusted the OR for other variables in a multivariate binary logistic regression model and found that ASA was still associated with an increased OR (OR: 1.29, 95% CI: 1.01–1.65, *P* = 0.042).

**Figure 2:**
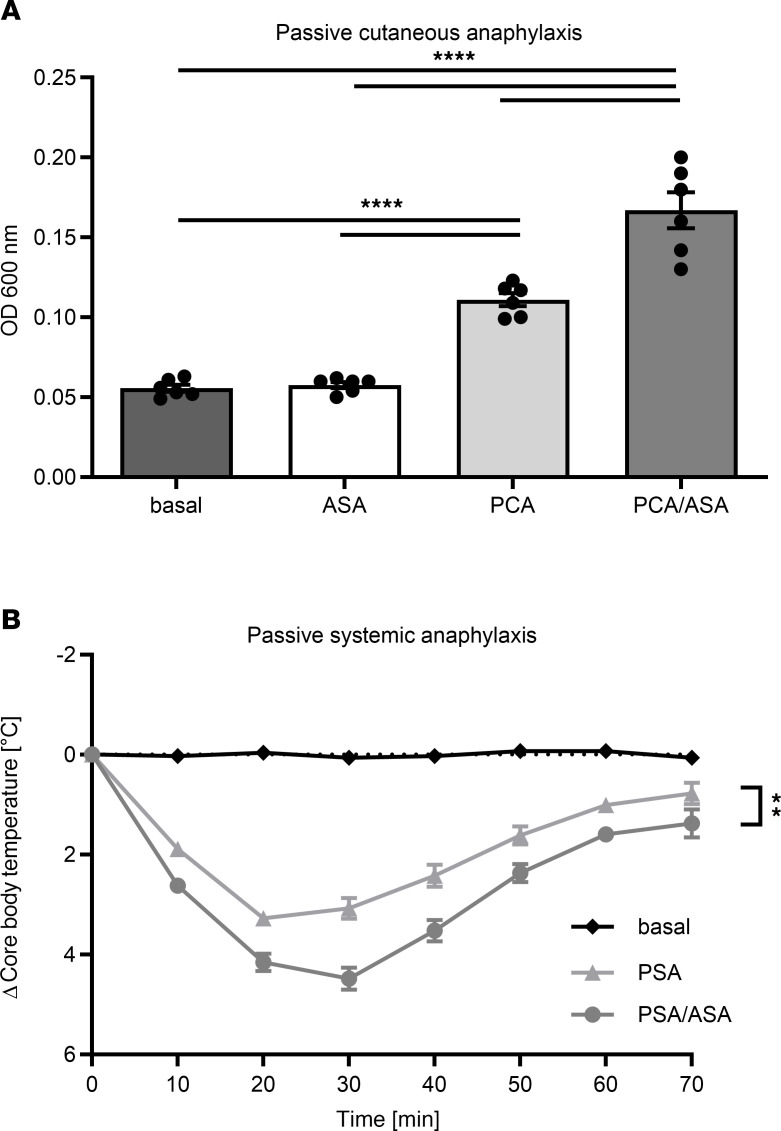
ASA enhances the severity of anaphylaxis. BL6 mice were pretreated with ASA or vehicle prior to (**A**) PCA or (**B**) PSA anaphylaxis induction. Mice were then passively sensitized with anti–TNP IgE. Anaphylaxis was induced by injection of TNP-BSA. (**A**) In the PCA model, absorbance of Evans blue dye was measured as an indicator of anaphylaxis severity. Absorbance is shown as the arithmetical mean ± SEM (*n* = 6). ****P < 0.0001, by 1-way ANOVA with Tukey’s multiple-comparison test. (**B**) In the PSA model, the rectal temperature was measured, and the data show its development over time, represented as the arithmetical mean ± SEM (*n* = 17–23). **P < 0.01, by 2-tailed, unpaired *t* test of the AUC. Asterisks in **B** show a significant difference between the AUC between PSA and PSA/ASA.

To determine whether the role of ASA in anaphylaxis outcomes can also be observed in animal models, C57BL6 (referred to hereafter as BL6) mice were treated or not with ASA. We used passive cutaneous anaphylaxis (PCA) and passive systemic anaphylaxis (PSA) models. Anaphylaxis induced with anti-trinitrophenol IgE (anti–TNP IgE) was significantly increased in ASA-treated mice (Figure 2A). Similarly, we observed a significantly increased drop in body temperature in ASA-treated versus untreated mice in the PSA model (Figure 2B). A comparable drop in body core temperature was observed with the use of COX1 and COX2 inhibitors in the PSA model (Supplemental Figure 1; supplemental material available online with this article; https://doi.org/10.1172/JCI175397DS1). BL6 mice were chosen for the largest part of this study because their anaphylactic reactions were stronger and their reaction outcomes more robust compared with the BALB/c mouse strain and the availability of KO strains on this background; this proanaphylactic effect of ASA was detected in both genotypes (Supplemental Figure 2).

### The anaphylaxis-promoting effect of ASA depends on effector cells, not on the mediator-responsive tissue.

**Figure 3:**
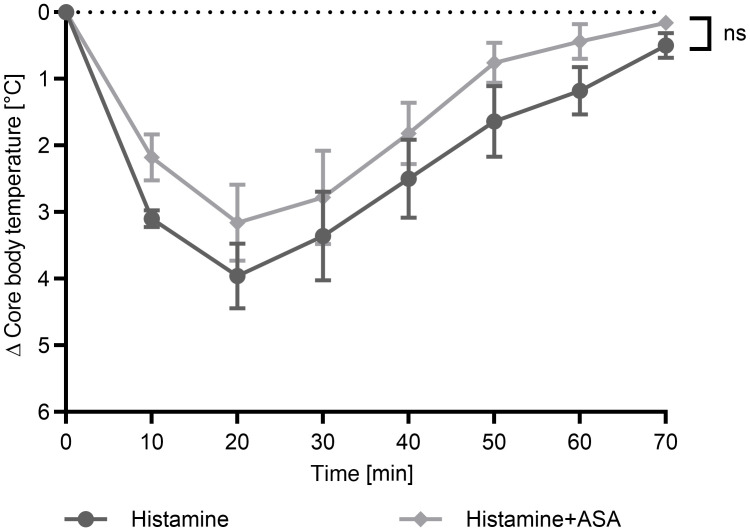
ASA has a negligible effect on the severity of histamine-induced anaphylaxis. Anaphylaxis was induced by direct histamine injection into the mice. Then, core body temperature was measured every 10 minutes. Data on core body temperature are shown as a function of time with the arithmetical mean ± SEM (*n* = 5). Statistical significance was determined by unpaired, 2-tailed *t* test of the AUC.

Having found that ASA promotes PSA, the question arose as to whether this effect is mediated by effector cells, so-called shock tissue (components responsive to MC mediators), or both. Histamine is the most critical mediator inducing hypothermia in the model (20–25). We therefore explored whether ASA has an effect on anaphylaxis when effector cells are bypassed by direct administration of histamine. However, we found that ASA did not significantly alter histamine-induced, MC-independent anaphylaxis (Figure 3). This suggests that the anaphylaxis-promoting effect of ASA occurred at the level of effector cells, rather than by potentiation of mediator effects at the end-organ level.

### Aggravation of anaphylaxis by ASA is PGE_2_ dependent.

**Figure 4:**
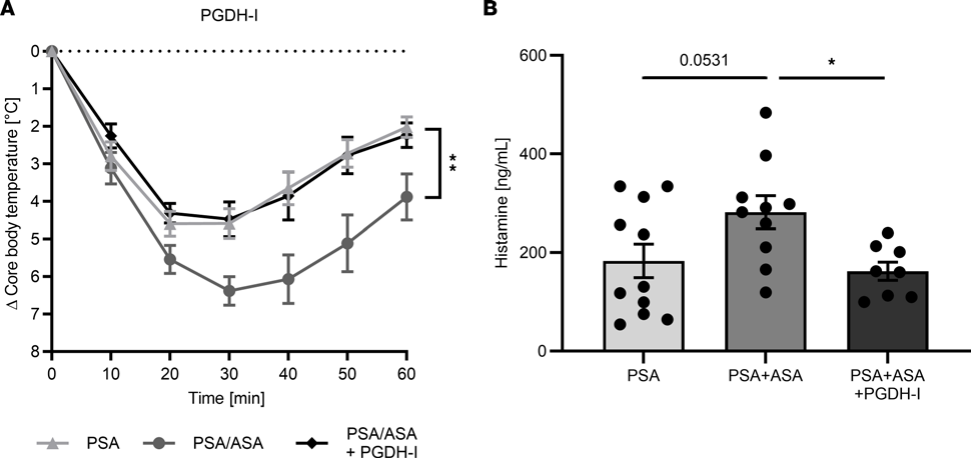
Stabilization of endogenous PGE_2_ counteracts the potentiation of anaphylaxis by COX inhibition. BL6 mice received ASA before induction of PSA by administration of TNP-specific IgE followed by TNP-BSA, as described in Figure 2B. (**A**) In 1 group of mice, the PGDH inhibitor was used 2.5 hours prior to elicitation of anaphylaxis. Data on core body temperature are shown as a function of time with the arithmetical mean ± SEM (*n* = 5–8). **P < 0.01, by 1-way ANOVA with Tukey’s multiple-comparison test, for PSA/ASA plus PGDH-I against PSA/ASA. (**B**) Serum histamine levels are shown as the arithmetical mean ± SEM (*n* = 8–11). *P < 0.05, by 1-way ANOVA with Dunnett’s multiple-comparison test, for PSA/ASA plus PGDH-I against PSA/ASA.

We previously reported that reduced systemic PGE_2_ levels are associated with anaphylaxis severity in the absence of COX inhibition (15). Therefore, we hypothesized that stabilizing PGE_2_ may counteract the increased severity of anaphylaxis in the context of ASA. Indeed, and as shown in Figure 4, the aggravation of anaphylaxis by ASA was reversed by prior application of a 15-hydroxyprostaglandin dehydrogenase (PGDH) inhibitor (PGDH-I), which interferes with PGE_2_ degradation, thereby confirming that a lower PGE_2_ level was the aggravating factor (Figure 4A). Systemic MC-dependent anaphylaxis is also reflected by an increase in systemic histamine, which is released after MC activation (5). The ameliorating effect of PGDH-I was related to MC activation, as systemic histamine levels decreased (*P* = 0.029) when mice were pretreated with ASA plus PGDH-I (ASA+PGDH-I) over ASA (PSA+ASA) alone (Figure 4B).

### EP2, 3, and 4 receptor agonists reverse the aggravation of anaphylaxis by ASA.

PGE_2_ acts via 4 different E-prostanoid (EP) receptors: EP1–4, however only EP2, EP3, and EP4 are ubiquitously expressed and active in the context of allergy and asthma. To better understand the role of each EP receptor in the aggravation of anaphylaxis by ASA, we administered different EP receptor agonists prior to PSA induction.

**Figure 5:**
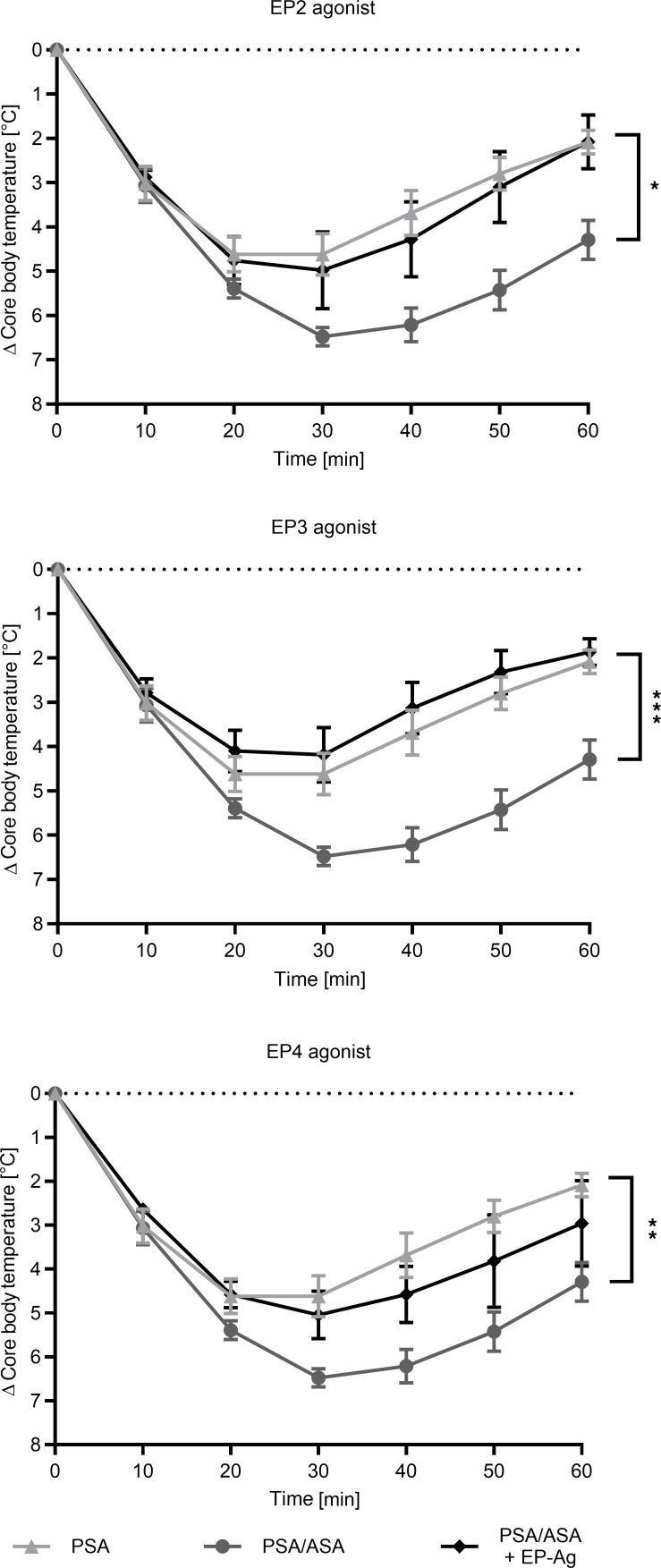
EP receptor agonists decrease the anaphylaxis severity caused by ASA. PSA was induced as described in Figure 3. Groups of mice were pretreated with the EP agonist (EP-Ag) ONO-AE1-259 (EP2), ONO-AE-248 (EP3), or ONO-AE1-329 (EP4) prior to being challenged as in Figure 2B. Data on core body temperature are shown as a function of time with the arithmetical mean ± SEM (*n* = 5–11). Asterisks show a significant difference between PSA/ASA and PSA/ASA+EP agonist: *P < 0.05 and ***P < 0.001, or PSA and PSA/ASA+EP agonist: **P < 0.01, by 1-way ANOVA with Tukey’s multiple-comparison test.

The EP4 agonist, and to a greater extent the EP2 agonist, partially alleviated ASA-induced aggravation, while the EP3 agonist completely reversed it, indicating that the concentration of the EP3 agonist used may be more effective than the EP2 and EP4 agonists at their respective concentrations (Figure 5).

### EP receptor–KO mice display altered anaphylaxis severity and reduced ASA-promoted aggravation.

Based on the results with EP agonists, we hypothesized that activation of either receptor was able to counter the ASA aggravation of anaphylaxis. To analyze this in further detail, mouse lines lacking the EP receptors 2, 3, or 4 were used. The different mouse strains retained the ability to undergo induced anaphylaxis in the absence of ASA (Supplemental Figure 3).

The important question was whether and to what extent the different genotypes respond to ASA with an aggravation of anaphylaxis. If any of the receptors operated on its own in protecting against anaphylaxis, the difference between ASA and baseline would be expected to vanish in the respective KO.

**Figure 6:**
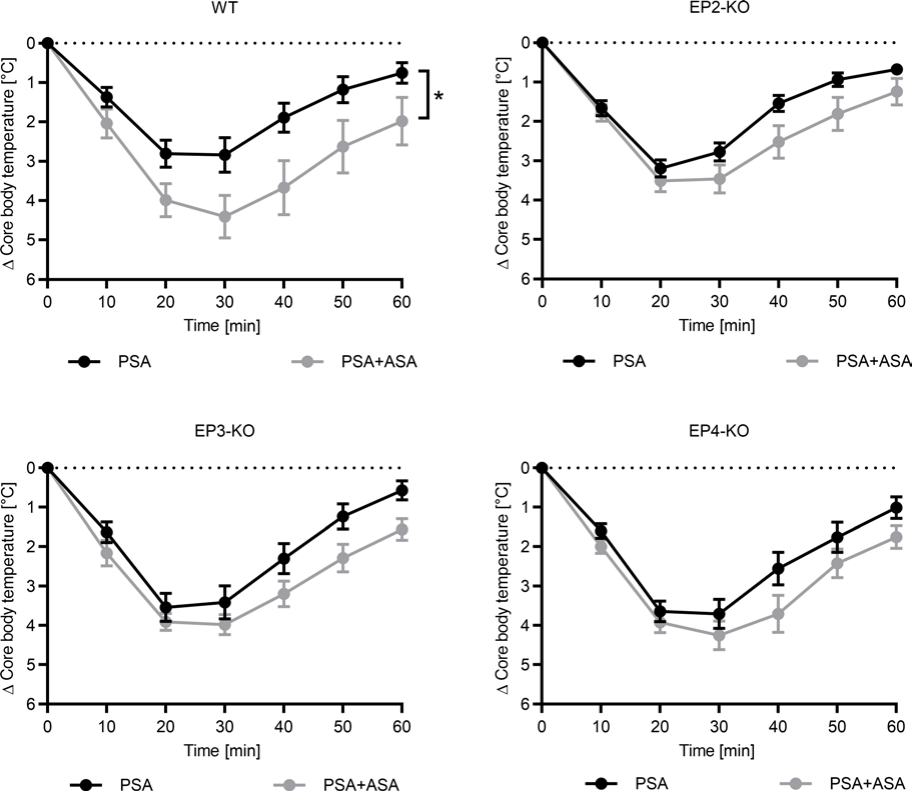
Anaphylaxis aggravation by ASA requires the operation of EP2, EP3, and EP4. BL6 mice (WT), EP2-KO mice, EP3-KO mice, and EP4-KO mice received only PBS (PSA) or ASA (PSA+ASA) before induction of PSA. PSA was performed as described in Figure 2B. Data on core body temperature are shown as a function of time with the arithmetical the mean ± SEM (*n* = 10–12). *P < 0.05, by 2-tailed, unpaired *t* test of the AUC.

We observed that ASA still had an anaphylaxis-promoting effect in EP2-, EP3- and EP4-deficient mice (Figure 6). However, the potentiation by ASA was clearly most pronounced in mice with an unperturbed EP repertoire, signifying that these mice were most strongly protected from anaphylaxis through all 3 receptors. The findings with EP agonists and receptor-deficient mice combined thus provide evidence that the protective role of PGE_2_ is most likely mediated by all 3 receptors.

### ASA aggravation operates via MCs, not basophils or histamine-responsive cells in humans.

**Figure 7:**
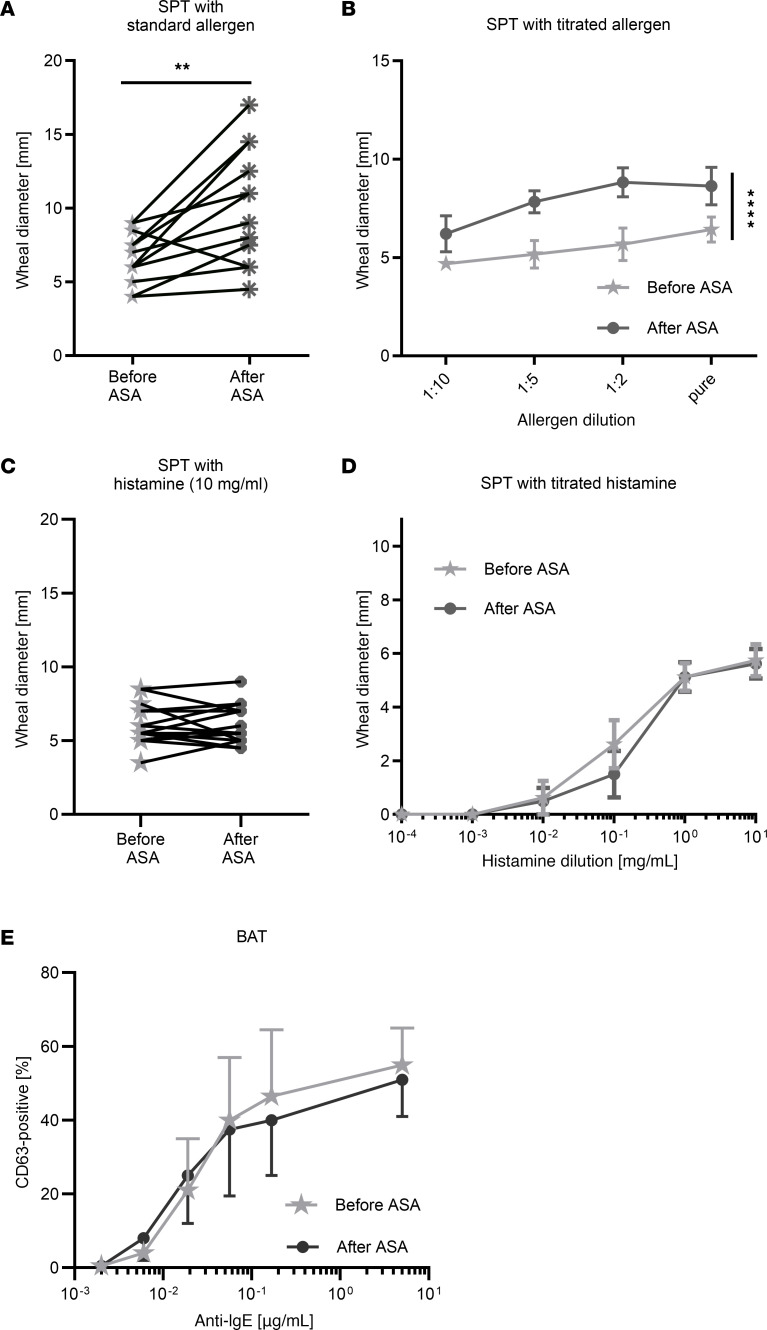
ASA treatment increases the propensity of MCs to degranulate in human skin in vivo. (**A**) Patients with allergy were subjected to standardized SPTs before and 24 hours after the last intake of ASA (300 mg orally for 3 consecutive days), and wheal size was measured. Each set of interconnected dots corresponds to 1 patient. (**B**) The allergen was applied at different concentrations of the pure allergen extract. The titrated SPTs are shown as the mean ± SEM (*n* = 7). (**C**) Histamine (at a standard dose of 10 mg/mL) served to induce wheals directly, bypassing MCs. (**D**) Histamine was applied in different concentrations. The SPTs with titrated histamine are shown as the mean ± SEM (*n* = 4). (**E**) Percentage of CD63^+^ basophils after stimulation with anti-IgE ex vivo in individuals treated with ASA as in **A**. Data are shown as the mean ± SEM (*n* = 7). **P < 0.01, by paired *t* test (**A** and **C**) and ****P < 0.0001, by unpaired *t* test of the AUC (**B**, **D**, and **E**).

Finally, we aimed to determine whether the ASA-mediated aggravation of allergic reactions is clinically detectable in patients with allergy (Figure 7). For this approach, we analyzed in vivo skin-prick test (SPT) reactivity to allergens in individuals with type 1 allergy. After undergoing a SPT, which leads to a local MC-dependent skin response (wheal) from allergen application in sensitized individuals, patients received 300 mg ASA orally for 3 days, and the SPT was repeated. Our results showed an increase in the wheal diameter in response to the allergen upon ASA treatment (Figure 7A). To learn whether the effect of ASA requires effector cells as intermediaries or results from the activity of PGE_2_ on the surrounding tissue, wheals were directly induced by histamine. In this control setting, MCs were circumvented by mediator application to the responsive components in the skin (e.g., endothelial cells, sensory nerves). Under this condition, we noted no effect of ASA on histamine dose response (Figure 7, C and D), confirming that any effect of ASA and PGE_2_ was occurring upstream and not at the end-organ level. This was equivalent to the results in the mouse, in which histamine-elicited anaphylaxis remained unaffected by ASA (Figure 3). To exclude the possibility that the effect of ASA is only detectable at the standard allergen concentration, we additionally performed a titrated SPT with higher allergen dilutions. At 1:2 and 1:5 dilutions, the wheal-supportive effects of ASA were even more pronounced (Figure 7B). The data highlight ASA’s potential to lower the threshold of skin MCs to become activated in their native surroundings, a phenomenon encountered in the majority of patients (Figure 7A). By virtue of high expression of FcεRI, basophils are another class of potential effectors during anaphylaxis. To explore whether basophils are altered in their responsiveness to FcεRI crosslinking by preceding ASA intake, we performed a basophil activation test (BAT) measuring the exteriorization of the activation marker CD63. Contrary to MCs, we observed that IgE-dependent activation of basophils ex vivo remained unchanged by prior ASA ingestion (Figure 7E). On the basis of these findings, we conclude that MCs are the primary effector cells that act as the sensors of PGE_2_. By reducing protective PGE_2_, ASA increased MC responsiveness to allergen, whereas basophil hyperactivity did not seem to contribute to anaphylaxis aggravation.

## Discussion

The aim of this study was to better understand the effect of ASA on anaphylaxis and to delineate the underlying mechanisms. We used in vivo mouse models and human allergy tests as well as epidemiologic data from the European Anaphylaxis Registry to address the question from different angles and to verify the findings by several approaches.

Data from the European Anaphylaxis Registry indicated an influence of ASA on anaphylaxis severity assessed by Brown’s severity grading, not only in the crude OR but also in the multivariate adjusted regression model. As a limitation, we could not adjust for cardiovascular diseases because of the association of cardiovascular diseases and ASA intake, but also age and the concomitant use of other medications.

All in all, the data from the European Anaphylaxis Registry in conjunction with the experimental mouse and human data support the notion that ASA is an aggravation factor of anaphylaxis. We unravel as the cause for this phenomenon a decrease in the production of PGE_2_. This is based on several findings. First, ASA as well as COX1 and COX2 inhibitors, aggravated murine anaphylaxis (Supplemental Figure 1). Second, stabilization of PGE_2_ reversed the effect of ASA. Third, agonists of PGE_2_ receptors likewise interfered with ASA-mediated aggravation. Fourth, EP receptor–KO mice displayed substantially weaker exacerbation by ASA than did their WT counterparts. In greater detail, PGE_2_ is normally degraded by PDGH. Inhibition of this enzyme blocks the oxidization of the 15-hydroxyl group of PGE_2_, an inactivating modification preventing further binding to EP receptors (26). Here, the strategy of PGE_2_ stabilization reversed the effect of ASA on anaphylaxis severity, confirming the critical role of PGE_2_. The observed effects of PDGH inhibition, and hence PGE_2_ stabilization, on anaphylaxis were confirmed at the level of systemic histamine measurements (Figure 4). In accordance with this finding, previous studies reported that histamine is a driver of anaphylaxis, as it lowers the core body temperature (23–25). When exogenously provided, histamine can mimic anaphylaxis in the absence of MC activation (23, 27–29), as replicated here.

Since a large body of literature has been published on MC modulation by PGE_2_ or its agonists (30), our aggregated data hinted that MCs are at the center of PGE_2_ activity. PGE_2_ predominantly acts via the G protein–coupled EP2-4 receptors. However, there are differences in signaling, since EP2 and EP4 couple to Gs proteins, while EP3 acts chiefly through Gi. Our data indicated that EP2 and EP4 agonists partially reversed the ASA-triggered aggravation of anaphylaxis. In fact, EP2 and EP4 and the subsequent increase in cAMP can directly interfere with MC activation (31–33). Consistently, in the context of allergic asthma, EP2 activation has likewise been found to inhibit lung MC and eosinophil activation (34, 35), whereas EP4 activation reduces allergen-induced airway hyperresponsiveness (36).

As shown in Figure 5, the EP2 agonist showed a slightly better ability to reverse the ASA-mediated anaphylaxis aggravation than did the EP4 agonist. This may be due to the more efficient functional coupling of EP2 to the cAMP pathway, as described by Machado-Carvalho et al. (37). Additionally, Torres-Atencio et al. reported a protective effect of PGE_2_ with MC degranulation through EP2 and EP4, which was established by the in vitro use of double-antagonist combinations targeting EP2, EP3, and EP4 (38). Collectively, there is sufficient evidence to suggest that EP2 and EP4 agonism can reduce anaphylaxis severity through direct suppression of MC activation in vivo. We further corroborated this by the use of EP2-KO and EP4-KO, which showed less aggravation with ASA than in WT BL6 mice, but the protection was not complete. This can be explained by assuming involvement of both receptors with partial redundancy between them. Taken together, although the PGE_2_/EP2 signaling axis is the best-understood pathway in MC inhibition (15, 31, 35, 39), EP4 can contribute in a similar way (38, 40–42), while displaying some differences in expression on MCs and downstream signaling (15, 43, 44).

Intriguingly, the EP3 agonist fully abolished the ASA-mediated aggravation, suggesting that at the concentration used in our study, the EP3 agonist was more potent than the EP2/EP4 agonists at their respective concentrations. This finding was somewhat unexpected, since PGE_2_ is known to activate MC degranulation through EP3 and has been described to counteract the EP2/PKA signaling pathway (40). Notwithstanding, the protective nature of EP3 could be verified in EP3-deficient mice. Interestingly, Kunikata et al. also reported on the antiallergic effects of an EP3 agonist, hinting that this effect may arise from the existence of different EP3 isoforms performing distinct functions (45). In fact, some EP3 isoforms can couple to Gs proteins, leading to a protective function like that of EP2 and EP4. The authors observed an EP3-dependent reduction of histamine and cysteinyl leukotriene release from lung explants challenged ex vivo with OVA. Since these are typical MC mediators (46), MCs could be identified as the eventual target regulating EP3-conferred protection in asthma and conjunctivitis (45, 47). The authors used MCs still embedded in a natural in vivo habitat, so there might be an indirect mechanism of EP3 agonists that suppresses MC activity in vivo. This could provide an additional or alternative explanation for the distinct EP3 isoforms discussed above, by which MC activation and anaphylaxis may be suppressed by EP3 agonism. In this context, bound EP3 receptors in the surrounding tissue may secrete MC inhibitors, which secondarily reduce MC activity and therefore downstream allergic responses. Since in our study ASA had no effect on allergic reactions induced by direct histamine injections in humans (by SPT) or mice (PSA-like model), MCs should thus be envisaged as the cells affected by a direct or indirect mechanism with regard to EP3 as well, in accordance with the study by Kunikata and colleagues (45). Basophils may also act as another cellular source of ASA-aggravated anaphylaxis, yet we could not detect altered ex vivo basophil activation following in vivo ASA pretreatment. In the literature, the addition of ASA or other NSAIDs has been suggested to aggravate anaphylaxis at the level of basophils; however, in these experiments, NSAIDs were added ex vivo to basophils (12, 13), whereas in our study, NSAID exposure occurred in vivo (i.e., administered to participants prior to having their blood drawn), which may explain the differences with our findings (12, 13).

In a recent publication, we compared serum PGE_2_ levels in 48 patients who had already experienced anaphylaxis (serum taken outside of the anaphylactic event) with that from 27 healthy controls (15). We showed that serum PGE_2_ levels were markedly reduced in patients with anaphylaxis. Furthermore, the PGE_2_ levels were inversely correlated with the severity grade of anaphylaxis. This is further evidence of the protective role of PGE_2_ in anaphylaxis and emphasizes that a paucity of PGE_2_, whether natural (15) or driven by ASA (this study), increases anaphylaxis susceptibility. In contrast to our previous results, another study has shown that plasma PGE_2_ concentrations do not differ significantly between patients at risk of food-induced anaphylaxis elicited by a lipid transfer protein (LTP) and healthy individuals (48). Given that our results were derived from patients who are prone to venom anaphylaxis (15), the genetic diversity of patients prone to food- or hymenoptera-induced anaphylaxis may explain these differences. Studies involving larger cohorts will be required to further clarify this aspect.

The data from human in vivo prick tests and ASA intake underpin the central role of PGE_2_ in in vivo MC reactivity and, therefore, anaphylaxis and hint at the potential therapeutic utility of the PGE_2_ network in altering severe anaphylaxis outcomes. A protective function of COX and PGE_2_ in dampening allergic symptoms has been suggested by other groups as well (45, 49, 50).

Taken together, we provide evidence of the connection between ASA and anaphylaxis based on epidemiological and in vivo and ex vivo mouse and human data and pinpoint PGE_2_ and its distinct receptors as the system involved. These findings are of high clinical relevance, as ASA is often used in older patients and frequently given in combination with cardiovascular drugs like beta blockers and ACE inhibitors, which have also been shown to enhance the risk for severe allergic reactions (6). As these drugs are frequently used simultaneously, they may increase the overall risk for severe or even fatal anaphylaxis in older patients, as indicated by the data from the European Anaphylaxis Registry, and should be considered more frequently in clinical practice. The therapeutic potential of molecules supporting the PGE_2_ pathway should be elucidated in more detail in future clinical trials.

## Methods

*Sex as a biological variable*. Our study exclusively examined female mice, as in our previous studies. It is unknown whether the findings are relevant for male mice.

*Mouse lines*. BL6 and BALB/c female WT mice (~10–12 weeks of age) were bought from Charles River Laboratories. The mice were housed under specific pathogen–free conditions in a temperature-controlled environment. Free access to standard chow and water was given.

*KO mouse strains*. KO mouse lines were generated as follows: EP2-KO mice were generated and described previously by Kennedy et al. (51). A targeting vector containing neomycin resistance and herpes simplex virus thymidine kinase genes driven by the mouse phosphoglycerate kinase promoter were used to disrupt the sequence of the targeted gene EP2 encoding the N-terminal of the protein. The construct was electroporated into 129S6/SvEv-derived (129-derived) TL-1 embryonic stem (ES) cells. Correctly targeted ES cells were injected into BL6 blastocysts. The resulting chimeric male animals were backcrossed with BL6 females. Simple sequence length polymorphism (SSLP) analysis confirmed that there were no chromosomal segments derived from 129, except for regions immediately flanking the disrupted locus proximal to chromosome 14. This mouse strain originated on a BL6;129 background and was backcrossed with BL6 mice for 5 generations (using a speed congenic protocol) to achieve homozygosity.

EP3-KO mice were newly generated in collaboration with Cyagen. The mouse *Ptger3* gene (GenBank accession number: NM_011196.3; Ensembl: ENSMUSG00000040016) is located on mouse chromosome 3. Three exons have been identified, with the ATG start codon in exon 1 and the TGA stop codon in exon 3. Exon 1 and exon 1 to exon 2 were selected as target sites, and 2 pairs of guide RNA–targeting (gRNA-targeting) vectors were constructed and confirmed by sequencing. *Cas9* mRNA and gRNA generated by in vitro transcription were coinjected into fertilized eggs to generate KO mice. F0 founder animals were identified by PCR followed by sequence analysis and bred with WT mice to test germline transmission and F1 animal generation. Heterozygous recombinant EP3-KO mice were crossed to generate homozygous KO mice, resulting in successful deletion of EP3 gene expression.

For EP4 KO, we used a tamoxifen-inducible (TMX-inducible) Cre mouse line (UBC-Cre, ERT2, B6. Cg-Tg (UBC-Cre/ERT2, 1Ejb/2J; The Jackson Laboratory), which ubiquitously expresses TMX-inducible Cre recombinase. In order to achieve the best possible degree of deletion, we titrated the concentration of i.p. administered TMX. Satisfying results were achieved with a daily dosage of 2.25 mg per mouse over 5 days, and EP4 gene expression was substantially reduced after TMX treatment compared with expression in vehicle-treated controls.

*PSA*. Anaphylaxis in mice was induced in a PSA model, which was also previously described (15). In brief, mice were treated with 50 mg/kg ASA or PBS, and 0.8 mg/kg TNP-IgE (BD Biosciences) was i.v. administered for passive sensitization. Afterwards, mice were given PGE_2_ (0.3–7.5 μg/kg), EP agonists (10 μg/kg), 15-PGDH inhibitor (10 mg/kg), or PBS 45 minutes (PGE_2_, EP agonists, PBS) or 2.5 hours (PGDH inhibitor) before induction of anaphylaxis with 0.14 mg/kg TNP-BSA (LGC Bioresearch Technologies). The severity of anaphylaxis was determined by measuring the rectal temperature every 10 minutes for 60 minutes (digital thermometer, Physitemp Instruments). Agonists for EP2 (ONO-AE1-259), EP3 (ONO-AE-248), and EP4 (ONO-AE1-329) were provided by ONO Pharmaceutical Co., Ltd.

Mouse strains with EP receptor KO were treated with ASA or PBS, and then anaphylaxis was induced and rectal temperatures measured as described above.

The COX1 (SC-560) and COX2 (celecoxib) inhibitors were administered (20 mg/kg) for 2 days. The last administration was given 24 hours or 16 hours before induction of anaphylaxis, as described above.

*Passive cutaneous anaphylaxis*. BL6 mice were treated with ASA or PBS and passively sensitized as described above. After this, 1% Evans blue in TNP-BSA was injected i.v. to induce anaphylaxis. Mice were euthanized, and vascular permeability was visualized with blue staining after 30 minutes. Skin samples were taken, and Evans blue dye was extracted with dimethylformamide (DMF). The concentration of Evans blue was quantified by measuring the OD at 600 nm.

*Serum histamine levels*. Serum histamine levels were measured using a previously described optimized autoanalyzer method (15). To adjust for interassay variation, the values were normalized so that the mean of all measurements was used as a common reference point.

*Histamine-induced anaphylaxis*. Mice were treated with 50 mg/kg ASA or PBS for 72 hours. On the following day, anaphylaxis was induced by i.v. injection of 4 mg/kg histamine into the tail vein.

*SPT*. Standardized SPTs were performed on patients with allergy or on healthy controls before ASA treatment and 24 hours after oral administration of 300 mg ASA for 3 consecutive days. One drop of (a) a test solution containing allergen extracts specific for the allergic patient or (b) histamine was applied on the forearm flexion side. Serial dilutions of allergen extract and histamine were used. The wheal diameter was measured 15 minutes after pricking. Histamine (10 mg/mL) was administered via skin prick as a control.

*BAT*. A BAT was conducted as described before (52). Briefly, heparinized whole blood from patients with allergy before and after ASA treatment was collected. Anti–human IgE was serial diluted in medium and incubated with the heparinized blood for 15 minutes at 37°C. Erythrocytes were lysed, and the remaining cells were fixed and stained for analysis with a MacsQuant Flow Cytometer (Miltenyi Biotec). Basophils were identified as CD3^–^CCR3^+^ cells; activated basophils were then detected on the basis of CD63 positivity (BD Biosciences).

*The EuropeanAnaphylaxis Registry*. The European Anaphylaxis Registry was established in 2007 as a real-world database to compile data on moderate and severe anaphylaxis. Specialized tertiary allergy centers in Germany, Austria, Switzerland, Poland, Italy, Spain, Ireland, Greece, France, Bulgaria, and Brazil report anaphylaxis cases with pseudonymized patient data. Data collected included symptoms, elicitors, cofactors, and treatment of the anaphylaxis. Informed consent from all patients was collected before entering data into the Registry. The study was registered with ClinicalTrials.gov (NCT05210543). Data until March 2024 were used for these analyses.

### Statistics

Statistical analyses were conducted with GraphPad Prism 8.4.3 (GraphPad Software). Comparisons of 2 groups were done using a 2-tailed *t* test. For 3 or more groups (e.g., histamine measurement), the data were compared using 1-way ANOVA. The differences of body temperatures in the PSA model were compared with a *t* test (2 groups) or 1-way ANOVA (3 or more groups) for the AUC. Data from the European Anaphylaxis Registry were analyzed using R, version 4.3.1 (16.06.2023) (53)

#### Multivariate binary logistic regression analysis.

To determine the influence of ASA on anaphylaxis severity in humans based on data from the European Anaphylaxis Registry, we calculated unadjusted ORs and conducted a multivariate binary logistic regression analysis. The dataframe was filtered to adults with a grade II or III reaction (Brown’s severity grading), which was also the dependent outcome variable. As independent variables, we chose baseline characteristics (patient’s age, male sex, elicitor venom [vs. non-venom-induced anaphylaxis]); concomitant ongoing diseases (allergic rhinitis, asthma, atopic dermatitis, cardiovascular disease, diabetes mellitus, infection, malignant disease, mastocytosis, [other] food allergies, nasal polyps, thyroid disease); concomitant intake of medication (ASA, ACE inhibitors, beta blocker, angiotensin 2 receptor antagonists, calcium antagonists, proton pump inhibitors, statins, thyroxine, diuretics); and cofactors (alcohol intake, emotional burden, physical exercise, menstruation, hormone therapy, sleep deprivation, acute infection). Since only complete observations were used, we excluded concomitant diabetes, concomitant (other) food allergy, concomitant nasal polyps, hormonal therapy, sleep deprivation, and menstruation because of too many missing values. Furthermore, we excluded cardiovascular disease, intake of thyroxin, and cofactor infection due to moderate-to-strong correlations with other independent variables. We reduced the number of slightly correlating items by excluding angiotensin 2 receptor antagonists, calcium antagonists, statins, and diuretics, which correlated with other concomitant medications. Age was included as a binary variable, in which the cutoff was determined with the median split approach (≤ median age vs. > median age). The final model included male sex, age above the median, venom-elicited reaction (vs. non-venom-elicited reaction), ongoing asthma, rhinitis, atopic dermatitis, infectious disease, malignant disease, mastocytosis, thyroid disease, intake of ACE inhibitors, intake of ASA, intake of beta blockers, intake of proton pump inhibitors, emotional burden, exercise, and alcohol consumption as independent predictive variables, and a stepwise predictor selection was conducted. The results of the model are reported as ORs and 95% CIs.

### Data availability

Data are available in public repositories (no large datasets were generated), in the Supporting Data Values file, or from the corresponding author upon request.

### Study approval

Animal studies were conducted with the approval of the local authorities (LaGeSo, Berlin, Germany: approval number G0004/16 and G0062/20). Human in vivo SPTs were conducted with the approval of the ethics committee at Charité-Universitätsmedizin Berlin (EA1/150/12). Data analysis from the European Anaphylaxis Registry was conducted with the approval of the ethics committee at Charité-Universitätsmedizin Berlin (EA1/079/06) and was accredited by the local ethics committees at the participating centers.

## Author contributions

MB and MW designed the research study. PM, VH, DMW, and MB conducted experiments, acquired data, and/or analyzed the data. All authors interpreted the data. MW, PG, and MB wrote the manuscript.

## Supplementary Material

Supplemental data

Supporting data values

## Version 1 03/03/2025

Electronic publication
