# Supplementary material for: Acetylsalicylic acid aggravates anaphylaxis in a PGE2-dependent manner
Source: J Clin Invest. 2025 Mar 3;135(5):e175397. doi: 10.1172/JCI175397 (PMC11870728; doi:10.1172/JCI175397)
Supplement: Supplemental data [file jci-135-175397-s048.pdf]

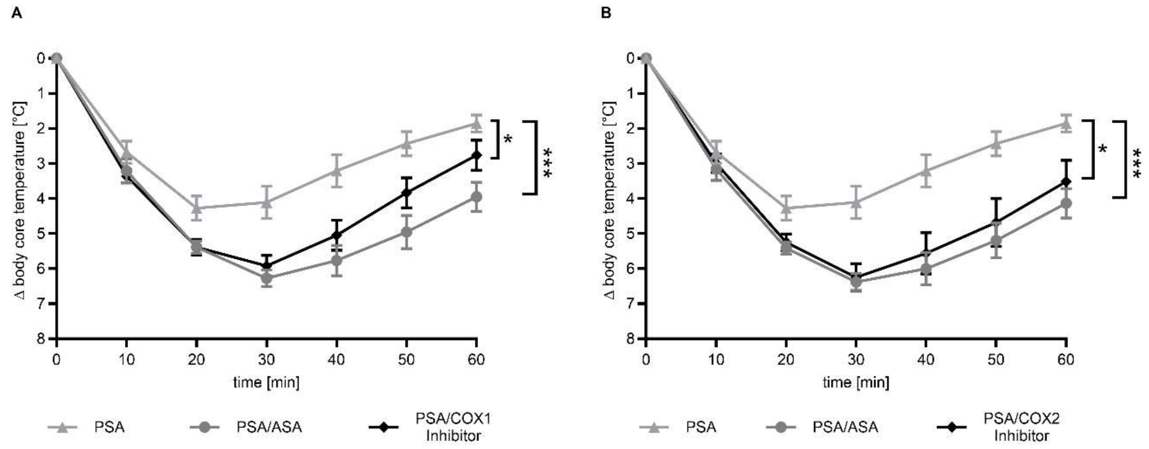

Figure S1. COX1 and COX2 inhibitors mimick ASA effect on anaphylaxis in the PSA model. BL6 mice were pretreated with ASA or a selective inhibitor of (A) COX1 (SC-560) or (B) COX2 (Celecoxib). Body temperatur change is represented as the arithmetical mean ± SEM (n=6-10). Statistical test: One-way ANOVA with Tukey's multiple comparison. Asterisks show a significant difference between the areas under the curve. * = $p < 0.05$; *** = $p < 0.001$

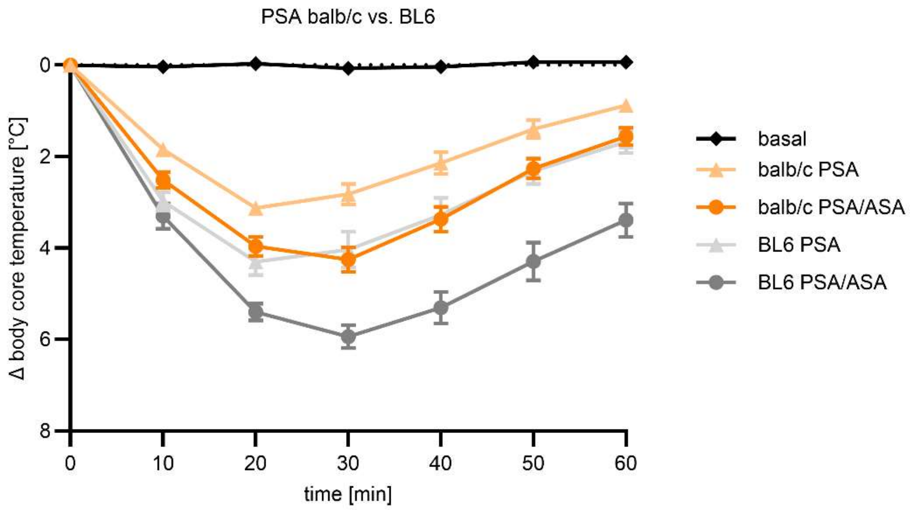

Figure S2. Comparison of rectal temperatures in the PSA in Balb/c and BL6 mice. Data shown as arithmetical mean ± SEM (at least n=14).

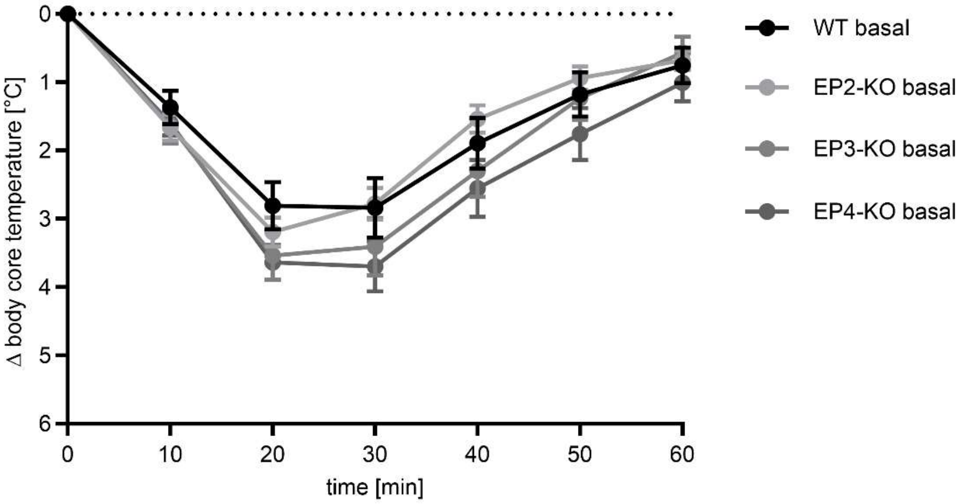

Figure S3. EP2, EP3, and EP4 knockout mice retain the ability to induce anaphylaxis at baseline, i.e., in the absence of ASA. Body core temperature change is represented as the arithmetical mean ± SEM (n=6-8). Statistical test: One way ANOVA with Tukey's multiple comparison of areas under the curve.
